# Immune-response 3′UTR alternative polyadenylation quantitative trait loci contribute to variation in human complex traits and diseases

**DOI:** 10.1038/s41467-023-44191-1

**Published:** 2023-12-15

**Authors:** Lei Li, Xuelian Ma, Ya Cui, Maxime Rotival, Wenyan Chen, Xudong Zou, Ruofan Ding, Yangmei Qin, Qixuan Wang, Lluis Quintana-Murci, Wei Li

**Affiliations:** 1 Institute of Systems and Physical Biology, Shenzhen Bay Laboratory, Shenzhen, 518055 China; 2 Division of Computational Biomedicine, Department of Biological Chemistry, School of Medicine, University of California, Irvine, CA 92697 USA; 3 Institut Pasteur, Université de Paris, CNRS UMR2000, Human Evolutionary Genetics Unit, F-75015 Paris, France; 4 Human Genomics and Evolution, Collège de France, F-75005 Paris, France

**Keywords:** Gene regulation, Computational biology and bioinformatics

## Abstract

Genome-wide association studies (GWASs) have identified thousands of non-coding variants that are associated with human complex traits and diseases. The analysis of such GWAS variants in different contexts and physiological states is essential for deciphering the regulatory mechanisms underlying human disease. Alternative polyadenylation (APA) is a key post-transcriptional modification for most human genes that substantially impacts upon cell behavior. Here, we mapped 9,493 3′-untranslated region APA quantitative trait loci in 18 human immune baseline cell types and 8 stimulation conditions (immune 3′aQTLs). Through the comparison between baseline and stimulation data, we observed the high responsiveness of 3′aQTLs to immune stimulation (response 3′aQTLs). Co-localization and mendelian randomization analyses of immune 3′aQTLs identified 678 genes where 3′aQTL are associated with variation in complex traits, 27.3% of which were derived from response 3′aQTLs. Overall, these analyses reveal the role of immune 3′aQTLs in the determination of complex traits, providing new insights into the regulatory mechanisms underlying disease etiologies.

## Abstract (web summary)

Alternative polyadenylation (APA) has a key role in the post-transcriptional regulation of most human genes but is understudied in cells of the immune system. Here, the authors construct an atlas of cell type-specific APA events in various immune cell-types and stimulation conditions, providing evidence of widespread stimulation-responsiveness and association with immune-related traits.

## Introduction

Genome-wide association studies (GWASs) have identified thousands of genetic variants associated with common traits and diseases in humans, and the majority (~90%) of these variants are located in non-coding regions^1–3^. Yet, understanding the molecular mechanisms through which these variants contribute to variation in physiological and pathological phenotypes remains challenging. Molecular quantitative trait loci (xQTL) analysis, which identifies associations between genetic loci and specific molecular traits, provides an essential link between genotype and phenotype and helps elucidate the functional effects of non-coding genetic variants. Population-scale studies have enabled the identification of a large variety of xQTLs, including expression QTLs (eQTLs)^4^, splicing QTLs^5^, 3′aQTLs^6^, DNA methylation QTLs^7^, DNA accessibility QTLs^8^, and RNA methylation (m6A) QTLs^9^.

Over the last decade, GWASs have largely increased our knowledge of the genetic architecture of human traits and diseases; yet, they do not provide context-specific information on cell types or environmental factors that affect specific disease risks and outcomes. Recent large-scale eQTL studies, such as Genotype-Tissue Expression (GTEx), have provided a rich repository for tissue-specific effects of disease risk variants, however, on average, only 20% of GWAS loci are associated with an eQTL in a disease-relevant tissue^4^. The poor performance of eQTL studies to identify disease-causing genes is partly due to the fact that they are generally performed on heterogeneous cell types, limiting the investigation of cell type-specific genetic effects in more relevant disease contexts^10^. Several other eQTL studies, such as those based on the Database of Immune Cell Expression (DICE), have been performed on homogeneous cell types from the same individuals and highlighted the role of cell type-specific eQTLs in mediating the effects of GWAS loci^11,12^^,^. In addition, many disease variants do only manifest in response to external stimuli, i.e., response eQTLs (reQTLs), wherein the eQTL effect differs between baseline and immune stimulation^13–16^. reQTLs can impact the transcriptional outcome following immune stimulation and highlight gene-by-environment interactions where the genetic effects are conditioned by the cellular environment. Still, although context-specific eQTLs can be informative for understanding disease-causing mechanisms, a large proportion of trait-associated non-coding variants^4^ remain unexplained.

Alternative polyadenylation (APA) plays an essential role during the post-transcriptional regulation of most human genes. By employing different polyadenylation (polyA) sites, genes can either shorten or extend 3′-untranslated regions (UTRs) that contain *cis*-regulatory elements, such as microRNAs or RNA-binding protein (RBP) binding sites^17^. APA can, therefore, affect the stability and translation efficiency of target mRNAs and the cellular localization of proteins. More importantly, our recent work identified ~0.4 million common genetic variants in GTEx associated with APA (3′aQTLs)^6^, which co-localize with ~16.1% of human traits and disease-associated loci. However, because GTEx does not provide information on cell types or cellular environments, the use of QTL analyses to explain the etiology of human disease-associated variants remains limited. Interestingly, APA plays a critical role in the immune system^18–20^. For example, widespread 3′UTR shortening is observed upon T cell activation^21^, and global 3′UTR shortening is observed during the innate immune response to bacterial infections, such as with *Listeria monocytogenes* and *Salmonella* *typhimurium*^22^, however, APA variations and contribution to intra- and inter-population differences in immune responses are poorly characterized.

Here, we sought to obtain insights into the genetic control of APA regulation, and used our DaPars2 algorithm^23^ to construct a harmonized atlas of cell type-specific human APA events from 18 immune cell types and 8 stimulation conditions. Using these datasets, we mapped 3′aQTLs and identified ~0.6 million common genetic variants associated with APA, corresponding to a total of 9,493 independent 3′aQTLs. In doing so, we provide the first evidence of widespread stimulation-responsive 3′aQTLs. Collectively, our results inform the genetic architecture of APA in immune cell types, both at the basal state and upon stimulation, and can be used to understand the regulatory bases of a significant proportion of genetic variants located in non-coding regions.

## Results

### Immune 3′aQTL atlas for 18 immune cell types and 8 stimulation conditions

**Fig. 1:**
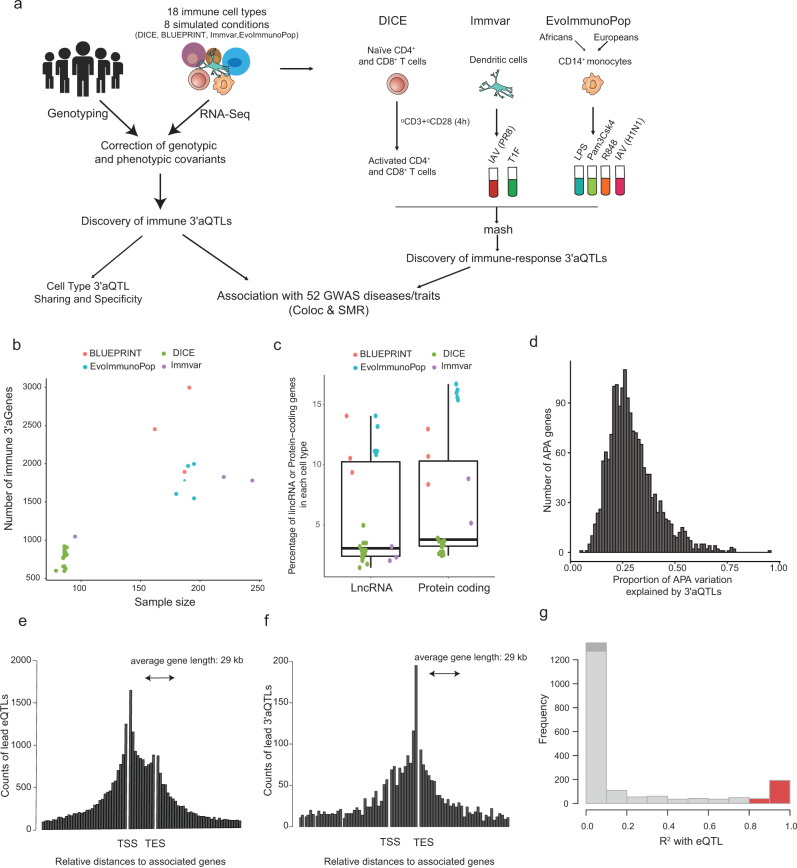
3′aQTL landscape across immune cell types. **a** Study design and analysis workflow. **b** Scatterplot showing the correlation between the number of identified 3′aGenes and sample size. **c** Percentage of lncRNAs (*n* = 341) and protein-coding genes (*n* = 13,001) detected in each immune cell type. The center horizontal lines of the box plot show the median values and the boxes span from the 25th to the 75th percentile. **d** Proportion of APA variation explained by cell type-specific 3′aQTLs. **e** Position distribution of immune eQTLs and **f** immune 3′aQTLs over their associated genes. **g** Plot showing the overlap between 3′aQTLs and eQTLs. eQTL mapping was performed on the same set of genes used for 3′aQTLs mapping. Red color represents the number of 3′aQTLs that overlapped with eQTLs (R^2^ $\ge 0.8$).

To investigate the dynamics of APA events in different cell types, we applied our DaPars2^6^ algorithm, in which APA was modeled separately for each possible 3’UTR region, to each of the following 4 population-scale RNA-seq datasets (Fig. 1a): (i) DICE^11^, which includes 13 immune baseline cells types and 2 activated cell types (CD4^+^ and CD8^+^ T cells) across 97 individuals; (ii) BLUEPRINT^12^, which contains 3 major immune baseline cell types (CD14^+^ monocytes, CD16^+^ neutrophils, and naïve CD4^+^ T cells) across 197 individuals; (iii) ImmVar^24^, which includes baseline monocyte-derived dendritic cells (MoDCs), and MoDCs stimulated with type 1 interferon (T1F), or an influenza A virus engineered to maximize the type 1 interferon-induced response (IAV-ΔNS1), across 243 individuals; and (iv) EvoImmunoPop^25^ which includes baseline primary monocytes and monocytes activated with 3 Toll-like receptors (TLR) ligands (LPS, Pam_3_CSK_4_, or R848) or influenza A virus (IAV H1N1) from 200 individuals of African and European ancestry. The percentage of distal polyA site usage index (PDUI), which was based on two polyA-site models and represents APA usage, was calculated for each transcript in each sample. We further used probabilistic estimation of expression residual (PEER)^26^ to adjust for the known and technical covariates of PDUI values.

By applying our 3′aQTL mapping^6^, we discovered 9,493 loci associated with APA across 18 immune baseline cell types and 8 simulated conditions, for a total of ~0.6 million common genetic variants (false discovery rate [FDR] < 5%) (Supplementary Data 1). The average calculated genomic inflation factor was 1.013 (with a minimum value of 0.9914 and a maximum value of 1.046) (Supplementary Fig. 1). The immune 3′aQTLs identified are publicly available (http://bioinfo.szbl.ac.cn/immune_aqtl). As expected, the number of genes with a 3′aQTL (3′aGenes) correlated with sample size, not cell type or stimulation condition (Fig. 1b; Supplementary Fig. 2). We also found that 46.1% of long non-coding RNAs (LncRNAs) and 54.2% of protein-coding genes presented a 3′aQTL in at least one cell type (Fig. 1c). A median of 3.1% of lncRNAs and 3.8% of protein-coding genes presented a 3′aQTL per cell type. We further used a linear mixed model implemented in the GCTA program^27^ to estimate the heritability of APA variation contributed by 3′aQTLs observed in purified immune cell types (immune 3′aQTLs) for each gene within a 1-Mb *cis* region. At the individual cell type level, 3′aQTLs explained between 33.1% and 37.6% of APA variation (Fig. 1d). We also analyzed the relative position of immune 3′aQTLs and eQTLs across their associated genes, and found that immune 3′aQTLs are distributed approximately symmetrically around the 3′UTR region, while eQTLs are positionally distributed within the transcriptional start site (TSS) region (Fig. 1e, f). Finally, we investigated whether any regulatory variant simultaneously correlated with both APA (3′aQTLs) and gene expression (eQTLs) on the same set of genes and found that only 11% of immune 3′aQTLs overlapped with an eQTL (Fig. 1g). Taken together, our analyses revealed thousands of immune 3′aQTLs across multiple baseline and stimulated cell conditions and significantly expanded the reported number of APA-associated genetic variants.

### Global patterns of 3′aQTL sharing and specificity across immune cell-types

**Fig. 2:**
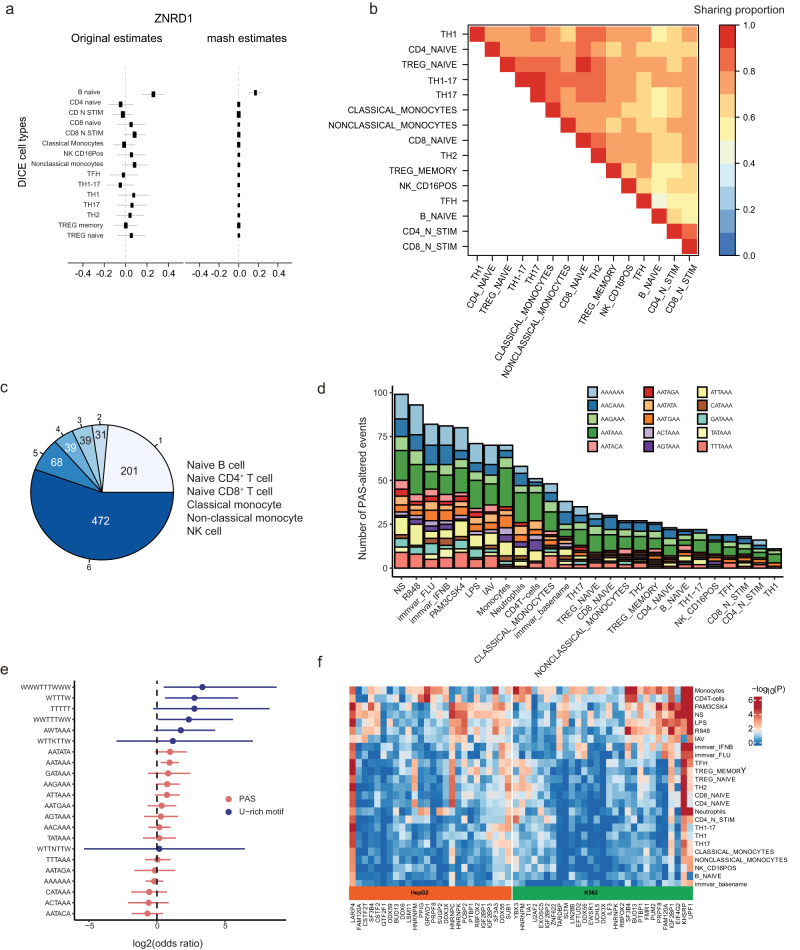
3′aQTL sharing and specificity across immune cell types in the DICE dataset. **a** The original estimates and mash estimates of 3′aQTL effect size for *ZNRD1*. The sample size (n) for each cell type varied from 79 to 88. The lines depict the median and the shadings the 95% confidence intervals (CIs). **b** Matrix showing pairwise sharing based on 3′aQTL effect size among all DICE immune cell types. For each pair of tissues, we considered the top 3′aQTLs that were significant (LFSR < 0.05) in at least one of the two tissues. The scale 0–1 on the right-hand side represents the proportion of these 3′aQTLs that were shared in magnitude—that is, that had effect estimates that were the same sign and whose sizes were within a factor of 2 of one another. The plot was clustered by ‘ward.D2’ implemented in hclust function in R. **c** Numbers of genes in which 3′aQTLs are shared among the six major immune cell types. The number outside the pie chart indicates the number of cell types where the 3′aQTL is active. **d** Summary of the PAS altered by 3′aQTLs across cell types. The *x*-axis shows the cell type, and the *y*-axis lists the number of 3′aQTLs that altered the PAS. **e** Enrichment of 3′aQTLs (*n* = 3,206) that altered PAS and uridylate-rich motifs and were proximal to polyA sites, compared with the rest of the genome. Data are presented as odds ratio. The lines depict the median and the shadings the 95% CIs. **f** Heatmap showing the 3′aQTLs significance for RBPs identified by ENCODE in each cell type by two-sided Fisher exact test. The bottom color bars represent the K562 and HepG2 cell lines. Values in the heatmap represent the degree of enrichment for 3′aQTLs in RBP binding peaks compared with the control.

To characterize the cell type-specificity of genetic effects in immune cells, we focused on the DICE dataset and sought to estimate the sharing of 3′aQTLs across immune cell types. We used the multivariate adaptive shrinkage method (mash) to re-estimate the 3′aQTL effect size by leveraging the correlation structure of QTL effect size across all cell types (Fig. 2a). For example, the 3′aQTL effect for *ZNRD1* estimates varied in sign and was modest except for a very strong signal in naïve B cells. Mash estimates could recognize the strong signal at this 3′aQTL, which outweighed the background information, and estimate a strong effect in naïve B cells with insignificant effects in other cell types. When clustering cell types based on the mash estimated value at the identified 3′aQTL, we found that non-stimulated cells are well-separated from stimulated cells (Fig. 2b). We did not observe, however, a clear distinction between myeloid and lymphoid cell types, suggesting that the genetic control of APA is largely independent of commitment to a specific lineage. Based on these results, we estimated the frequency at which the same 3′aQTLs are detected among the six major cell types in the DICE dataset (naïve B cell, naïve CD4^+^ T cells, naïve CD8^+^ T cells, NK cell, classical monocyte, and non-classical monocytes). We found that 55.5% of genes with a lead 3′aQTL were shared (local false sign rate [LFSR] <0.05) across the six major cell types (Fig. 2c), suggesting that the genetic effects on APA regulation are, in most cases, the same across immune cell populations. Furthermore, we observed that 378 immune 3′aQTLs are specific for certain immune cell groups, as was the case for 3′aQTL rs59377167 for *SRSF1*, rs28498977 for *YLPM1*, and rs2579396 for *ANKRD44*, which were detected only in adaptive immune cell types (Supplementary Data 2). Nevertheless, we also found that close to 25% of 3′aQTLs are cell type-specific, such as 3′aQTL rs2853231 for gene *UBR5*, which is specifically to naïve B cells. Collectively, we found that 3′aQTLs effects are predominantly shared across cell types, while observing a small number of cell-type-specific 3′aQTLs.

### Immune 3′aQTLs alter polyA motif and RNA binding sites

Next, we investigated the potential mechanisms through which immune 3′aQTLs contribute to APA events. We systematically examined the prevalence of polyA motif-altered immune 3’aQTLs across different cell types. In total, we identified 1,167 3′aQTLs associated variants that had the potential to alter polyA motifs and influence alternative 3′UTR usage of associated genes (Fig. 2d). We also investigated the enrichment of polyA-altered 3′aQTLs and AU-rich elements and found that AU-rich elements were strongly enriched among 3′aQTLs, as well as canonical AAUAAA motifs to a lesser extent (Fig. 2e). For example, we report a SNP (rs6852042) where a change from the reference allele T to the alternative allele A impaired the canonical polyA motif AAUAAA, leading to strong APA usage change upon immune stimulation but not in unstimulated cells (Supplementary Fig. 3). APA is also regulated by RBPs^6^. To validate the functionality of our detected 3′aQTLs and identify RBP regulators, we analyzed the correlation between 3′aQTLs and RBP binding sites by enrichment analysis. Only significantly associated 3′aQTLs located inside the gene body region were selected. RBP binding sites were defined as the significant CLIP-seq peaks, and the data for 162 RBPs were obtained from two ENCODE cell lines (HepG2 and K562). A set of randomly shuffled 3′aQTLs limited inside the gene body region and within the same chromosome was generated for comparison. We found 26 RBP regulators in HepG2 cell lines and 29 RBPs in K562 cell lines significantly enriched with 3′aQTLs (Fig. 2f). Among them, several polyadenylation and splicing factors were identified, and the recently experimentally validated LARP4 was also identified^6^. Collectively, the results of our analyses suggest that detectable APA events result from the alteration of polyA motifs and RNA binding sites in thousands of 3′aQTLs.

### Immune stimuli, rather than genetic ancestry, primarily drive condition-specific APA variation

**Fig. 3:**
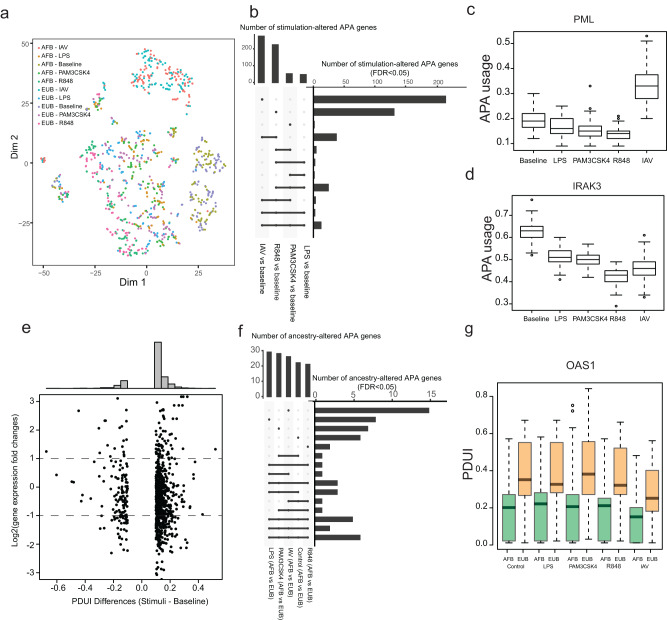
Stimuli, rather than genetic ancestry, primarily drive condition-specific APA variations. **a** t-SNE plot of APA usages for the five stimulation conditions in Africans (AFB) and Europeans (EUB) of the EvoImmunoPop cohort. Principal component analysis (PCA) was used as the unsupervised clustering method in the initial step before t-SNE. **b** Number of immune-stimulated APA genes compared to control. **c** Stimulus-specific APA usage for the *PML* gene. **d** Plot showing stimulus-specific APA usage for the *IRAK3* gene. **e** Plot showing the correlation between PDUI and gene expression changes upon stimulation. **f** Number of ancestry-altered APA genes across control and four different stimulation conditions compared between AFB and EUB cohorts. **g** 3′UTR lengthening effects across different conditions for the *OAS1* gene in African and European populations. For (**c**), (**d**) and (**g**), the sample size (n) for each cell type varied from 180 to 195, and the center horizontal lines of the box plot show the median values and the boxes span from the 25th to the 75th percentile.

To investigate the role of APA as a mechanism of local adaptation of different human populations, we next focused on the EvoImmunoPop cohort and searched for APA genes that have differential 3′UTR usages between African- and European-descent individuals. Unsupervised clustering revealed that samples tend to group by stimulation condition rather than by population (Fig. 3a), consistent with the idea that the response to immune stimulation is generally conserved across populations. Focusing on genes that changed their 3′UTR usage upon different stimulations, we identified a large number of differentially regulated APA genes for at least one condition (FDR < 5%) (Fig. 3b; Supplementary Fig. 4; Supplementary Data 3), with most APA genes showing differential usages in a stimulus-specific manner. Notably, the response to viral challenges (R848 and IAV) led to the strongest APA changes. We observed that *PML*, which encodes the promyelocytic leukemia protein and is involved in host antiviral defense^28^ (Fig. 3c), had significantly longer 3′UTR usage following IAV stimulation with respect to other stimuli. A few other genes also presented differential APA usage upon immune stimulation, such as *IRAK3*, which functions as a negative regulator of Toll-like receptor (TLR) signaling^29^ (Fig. 3d).

We further analyzed the APA usage in baseline and eight stimulation conditions and observed striking 3′UTR shortening events upon stimulation compared to baseline conditions (Supplementary Figs. 5–6). We subsequently analyzed whether differentially regulated APA genes exhibited expression changes by correlating PDUI differences with fold-change gene expression values. We found that most genes that undergo APA modifications upon immune activation only displayed moderate changes of their RNA levels (Fig. 3e), further highlighting the importance of studying the role of APA in the context of immune function. In addition, only 62 APA genes were differentially regulated between African- and European-descent individuals (FDR < 5%; Fig. 3f). Among these genes, we found that *OAS1* (Fig. 3g), which is involved in the antiviral innate immune response, had a longer 3′UTR in Europeans than in Africans. Incidentally, 88–96% of the differences in 3′UTR usage at *OAS1* could be explained by the rs10774671 allele, located on a Neanderthal-inherited haplotype that has been previously shown to be adaptively introgressed in European populations and protective against severe COVID-19^30,31^. Taken together, we found that while different stimuli, rather than genetic ancestry, are the main drivers of APA variation, differences in ancestry-related APA can also contribute to population differences in disease susceptibility.

### Immune stimulation drives robust response 3′aQTL

**Fig. 4:**
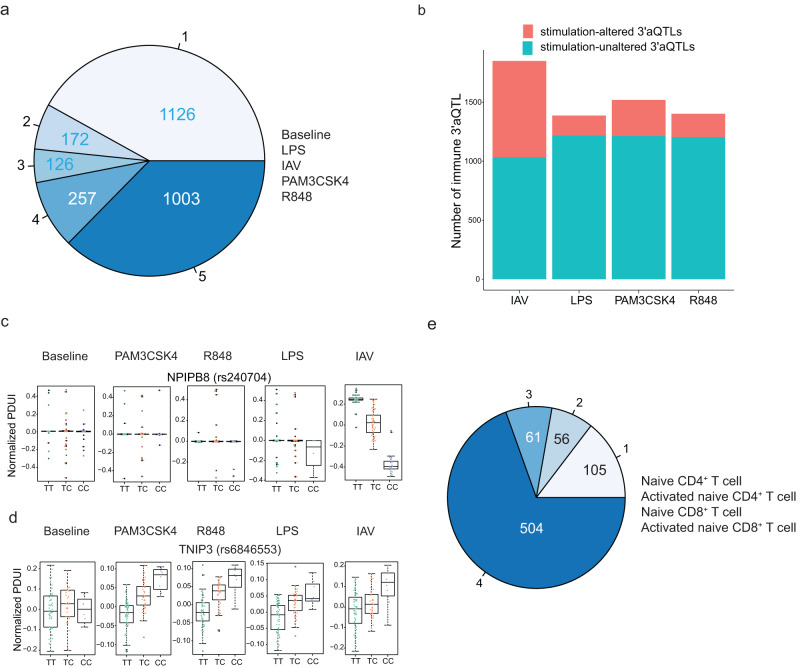
Immune stimulation induces robust response 3′aQTLs. **a** Numbers of 3′aQTLs shared between baseline and the four stimulated conditions in the EvoImmunoPop dataset based on mash analysis. **b** Distribution of response 3′aQTLs and shared 3′aQTLs upon immune activation. **c** PDUI values plotted by genotype for rs240704 (*NPIPB*) and (**d**) rs6846553 (*TNIP3*) across stimulation conditions. For (**c**) and (**d**), the center horizontal lines of the box plot show the median values and the boxes span from the 25th to the 75th percentile (*n* = 200). **e** The estimated number of cell types in which 3′aQTLs are inferred to be shared across T cells from the DICE dataset based on mash analysis.

To further investigate the stimulus-specificity of 3′aQTLs, we first analyzed the effect size estimates of the lead 3′aQTL for each gene and assessed the sharing of these 3′aQTLs across the various stimuli used in the EvoImmunoPop study^25^. Among the genes with at least one 3′aQTL (LFSR < 0.05), we estimated that 37.3% of 3′aQTLs were shared between baseline and the four stimulated conditions (Fig. 4a), indicating that a large proportion of 3′aQTL were stimulus dependent. Comparing each stimulus to the non-stimulated state, we estimated that an average of 28.3% of 3′aQTLs were either induced or lost upon immune stimulation (Fig. 4b), with 501, 431, 443, and 377 genes presenting a 3′aQTL specific for IAV, LPS, Pam_3_CSK_4_, and R848 stimulations, respectively (i.e., response 3′aQTLs; Supplementary Data 4). Among these, the variant rs240704 for *NPIPB8* had a strong response 3′aQTL upon IAV infection (Fig. 4c), and the variant rs6846553 for *TNIP3*, an inhibitor of NF-κB activation, showed genotype-dependent effects for all stimulation conditions but not at baseline level (Fig. 4d).

We next explored the presence of response 3′aQTLs in the other datasets. In dendritic cells, 51 genes had 3′aQTLs specific for IAV infection, and 27 genes had 3′aQTLs specific for T1F stimulation (Supplementary Data 5). The 23 genes that shared response 3′aQTLs among all stimulations were enriched in interleukin-12-mediated signaling pathway (*P* = 0.0012), neutrophil activation involved in immune response (*P* = 0.0017), and cytokine-mediated signaling pathway (*P* = 0.0043). Notably, *GSTO1* encodes glutathione S-transferase omega-1, and the rs45441599 variant, which regulates APA of *GSTO1*, was the most significant response 3′aQTLs in both IAV and T1F-stimulated cells. A similar trend was observed in T cells (Fig. 4e; Supplementary Data 6), with 31% of 3′aQTLs either induced or lost upon T cell activation. Interestingly, T cells and dendritic cells shared more response 3′aQTLs across stimulation conditions compared to monocytes. Taken together, we observed a large proportion of responsive 3′aQTLs in activated immune cells.

### Co-localization analysis identifies additional trait-associated 3′aGenes

**Fig. 5:**
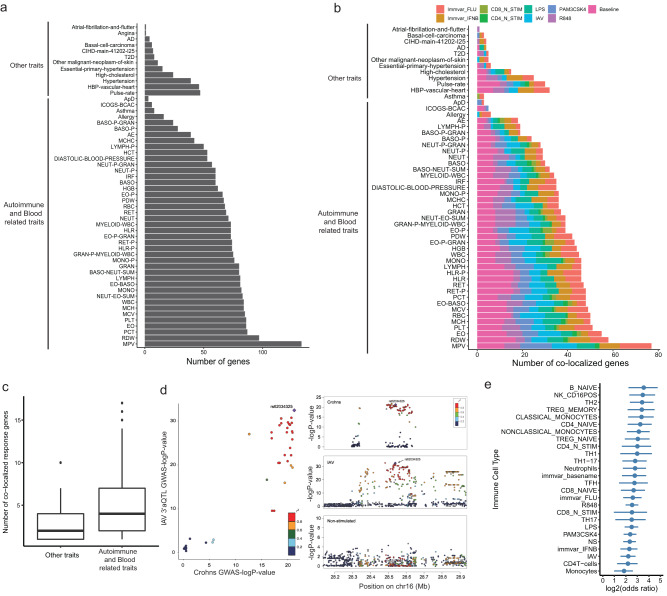
Co-localization analysis of 3′aQTLs and GWAS loci identifies additional trait-associated 3′aGenes. **a** Distribution of the number of trait-associated 3′aQTLs across 52 common traits based on co-localization analysis. **b** Distribution of the number of trait-associated response 3′aQTLs across 52 common traits based on co-localization analysis. **c** Comparison of the number of co-localized response 3′aGenes for autoimmune and blood-related traits with other traits (*n* = 23). The center horizontal lines of the box plot show the median values and the boxes span from the 25th to the 75th percentile. **d** Each dot represents a variant and is colored according to its LD to the selected response 3′aQTLs rs62034325. A bona fide colocalization signal should form a single spike toward the top right corner, as illustrated by colocalization between *NPIPB9* 3′aQTLs in IAV stimulated condition and Crohn’s disease. The details of the statistical test can be found in the “Methods” section. **e** Enrichment of disease-associated 3′aQTLs among 3′aQTLs (n for each cell type varying from 18,918 to 19,106) located <50 bp away from upstream or downstream polyA sites. Data are presented as odds ratio. The lines depict the median and the shadings the 95% CIs.

GWASs do not provide context-specific information on cell types or environmental factors that affect specific disease risks and outcomes. We hypothesized that cell type- and stimulus-specific 3′aQTLs could help interpret the underlying mechanisms at non-coding GWAS loci. We thus investigated the extent to which 3′aQTLs share the same putative causal variants with trait-associated variants. To do so, we compiled a set of well-powered GWAS summary statistics for 52 common human diseases and traits, including 40 autoimmune and blood-related traits and 12 other traits collected from previously published literature (Supplementary Data 7). We performed co-localization analysis using the coloc method^32^ and identified 2,912 3′aQTLs that co-localized with GWAS loci (PP4$\ge$0.75; Supplementary Data 8). In total, 9.1% of GWAS loci co-localized with a cell type-specific 3′aQTL. Among co-localized 3′aGenes, we identified *CD226*, whose 3′aQTL strongly co-localized with seven immune-related trait variants (PP4 = 0.97). As expected, we noticed that 3′aGenes were significantly linked to autoimmune and blood-related traits (*P* = 1.098 × 10^−8^), with respect to non-immune traits (Fig. 5a). We also cross-referenced these co-localized 3′aQTLs with co-localized eQTLs for each trait, and found that the majority of traits co-localized 3′aQTL are not eQTL. This observation is consistent with our recent findings^6^ that co-localized 3′aQTLs are largely distinct from eQTLs.

To further investigate the contribution of response 3′aQTLs to disease susceptibility, we performed co-localization analysis only on stimulated data. We found a substantial expansion of trait-associated genes upon immune stimulation (Fig. 5b), and these genes were largely associated with autoimmune- and blood-related traits (*P* = 3.145 × 10^−15^) (Fig. 5c). For example, we identified response 3′aQTLs for *ERAP2*, which were co-localized with Crohn’s disease for the CD4T-cells and R848 stimulation conditions (Supplementary Fig. 7). Variants associated with changes in *ERAP2* isoforms have previously been shown to increase Crohn’s disease risk upon immune stimulation^24^. Interestingly, we also found response 3′aQTLs for genes like *NPIPB9*, which only strongly colocalized with Crohn’s disease under IAV stimulation conditions (Fig. 5d). We also analyzed the immune disease-associated 3′aQTLs and found they are significantly enriched in regions that located <50 bp away from the upstream or downstream polyA sites (Fig. 5e). Collectively, our analysis revealed that 3′aQTL largely overlap with GWAS loci, with response 3′aQTLs encompassing a distinct group of disease-associated genes.

### Mendelian randomization analysis identifies trait-associated 3′aGenes

**Fig. 6:**
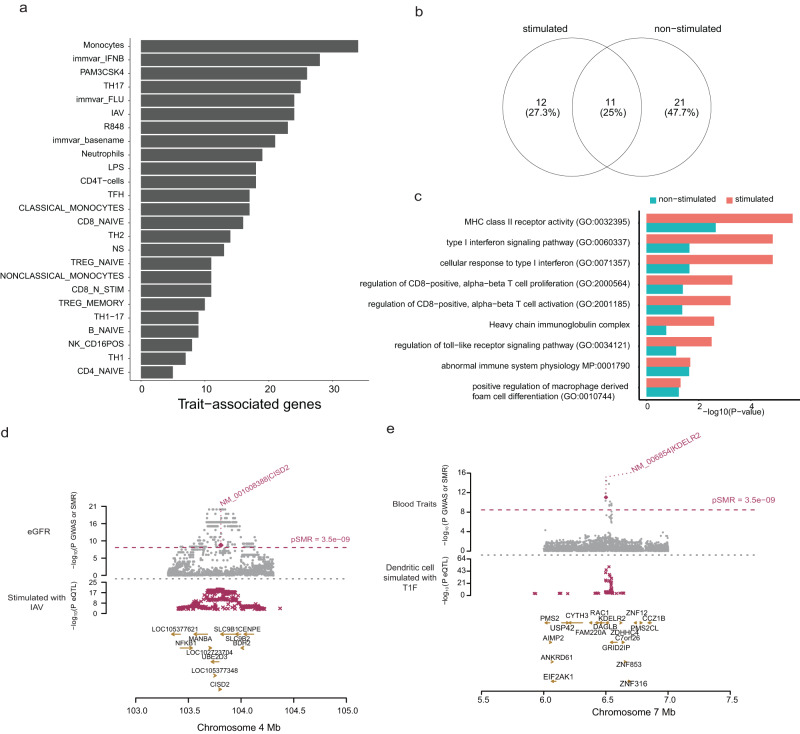
SMR analysis identifies trait-associated 3′aGenes. **a** Distribution of the number of significant trait-associated 3′aGenes across different cell types and stimulation conditions based on SMR analysis. **b** Comparison of trait-associated 3′aGenes in stimulated and non-stimulated conditions. **c** Functional gene ontology enrichment analysis of 3′aGenes for stimulated and non-stimulated conditions by two-sided Fisher exact test. **d** Identification of candidate 3′aGenes near the *CISD2* locus that have a potential pleiotropic/causal association with eGFR using data from dendritic cells stimulated with IAV (ImmVar). **e** Identification of candidate 3′aGenes near *KDELR2* locus that have a potential pleiotropic/causal association with a blood trait (MPV) using data from dendritic cells stimulated with T1F (ImmVar). For (d) and (e): We conducted using χ^2^ test. Top plot: gray points represent -log10(P values) for GWAS SNPs, and magenta rhombuses represent -log10(P values) calculated from the SMR analysis. Middle plot: magenta symbols represent -log10(P values) for 3′aQTLs. Bottom plot: gene locations.

To further prioritize APA events that may play a causal role in determining disease-risk and complex traits, we used a summary data-based Mendelian randomization (SMR) approach^33^ to test for significant genetic correlation between APA and GWAS loci. SMR analysis uses summary-level GWAS data to identify variants that associate with the APA usage of a gene and a complex trait of interest, suggesting either a pleiotropic or causal relationship. SMR analysis integrating these GWAS and 3′aQTLs data identified 37 3′aGenes that had pleiotropic effects or were potentially causal for GWAS diseases and traits (Fig. 6a; Supplementary Data 9). Taken together, co-localization and SMR analyses of immune 3′aQTLs identified 678 genes where 3′aQTL are associated with complex traits. When comparing the SMR and colocalization results, we found that although SMR detected 3′aQTL-associated genes significantly overlap with colocalized loci (*P* = 7.59 × 10^−16^, hypergeometric test), only 2.7% of colocalized signals are identified by SMR as putatively causal, highlighting the value of the SMR approach for gene prioritization. We then compared the set of 3′aGenes detected in the 8 stimulated conditions with 3′aGenes from other non-stimulated conditions and found that 27.3% of all trait-associated 3′aGenes were detected only in stimulated conditions (Fig. 6b), indicating that stimulation-specific 3′aGenes are more likely to contribute to variation in complex traits.

Functional enrichment analysis of stimulation-specific 3′aGenes revealed that they were highly enriched for several immune response-related GO terms, including regulation of CD8-positive, alpha-beta T cell activation (*P* = 5.12 × 10^−4^) and cellular response to type I interferon (*P* = 1.44 × 10^−5^) (Fig. 6c). However, we did not observe any relevant pathways for non-stimulated genes. Our SMR analysis identified several interesting response 3′aGenes that are potentially causally associated with traits. For example, SMR detected *CISD2* as a response-associated 3′aGene associated with the estimated glomerular filtration rate (eGFR) GWAS trait (*P*_SMR_ = 2.49 × 10^−15^) (Fig. 6d). In addition, we identified *KDELR2* as response 3′aGenes influencing blood immune traits (*P*_SMR_ = 1.20 × 10^−16^, respectively) (Fig. 6e). *KDELR2* is unfolded protein response gene and involved in multiple important cellular functions such as regulating ER stress, cell proliferation, and immune response^34^. Taken together, the identification of putatively causal cell type-specific response 3′aQTLs that co-localize with GWAS loci may help elucidate the mechanism through which genetic variation may contribute to disease pathophysiology.

## Discussion

The human immune system plays a key role not only in host defense against pathogenic agents but also in autoimmune and inflammatory diseases, cancer, metabolism, and aging. In light of this central role in a large variety of many human pathologies, it is crucial to understand the natural levels of variability of immune responses at the population level and their relation to disease susceptibility or progression. Investigating the role of genetic variation on the immune response is challenged by the complexity of the immune system, which consists of many different cell types that respond to a plethora of signals by activating intricate signaling cascades with diverse kinetics. With increasing evidence to suggest that APA regulation has an essential role in driving immune response variation, there is a strong need to characterize the genetic basis of APA events. Therefore, we developed an atlas of immune 3′aQTLs and response 3′aQTLs using a diverse set of immune cell populations and stimuli. We found these immune 3′aQTLs can explain 33.1–37.6% of APA variation, higher than the proportions we observed in GTEx data. Comparing with immune eQTLs, 3′aQTLs are distributed around 3′UTR region. We also demonstrated that condition-specific APA variation is primarily driven by immune stimuli rather than genetic ancestry. More importantly, we observed extensive response 3′aQTLs upon immune stimulations, and these response 3′aQTLs colocalized with a distinct group of disease variants. We observed that many 3′aQTLs colocalized with various blood cell count traits suggesting 3′aQTL may affect the level of blood cell counts. In doing so, our study provides new insights into the role of APA in the diversity of human immune system, and presents a powerful resource to map genetic variants to cell- and context-specific functional regions genome-wide and, therefore, to their context-specific effects on disease phenotypes.

## Methods

### Datasets

We analyzed the transcriptome data from 13 immune cell types across 91 individuals^11^ from the DICE dataset. These cell types included three innate immune cell types (CD14^high^CD16^—^ classical monocytes, CD14^—^CD16^+^ non-classical monocytes, and CD56^dim^CD16^+^ NK cells), four adaptive immune cell types that have not encountered cognate antigen in the periphery (naïve B cells, naïve CD4^+^ T cells, naïve CD8^+^ T cells, and naïve regulatory T cells [Tregs]), six CD4^+^ memory or more differentiated T cell subsets (Th1, Th1/17, Th17, Th2, follicular helper T cell [Tfh], and memory Tregs), and two activated cell types (naïve CD4^+^ and CD8^+^ T cells that were stimulated ex vivo). We also analyzed 563 samples from the ImmVar project^24^. These samples included monocyte-derived dendritic cells at rest and stimulated with influenza infection or type 1 interferon. In addition, we analyzed 970 samples from the EvoImmunoPop project^25^, which included 200 healthy male donors of self-reported African and European ancestry. The samples were collected from non-stimulated monocytes and monocytes exposed for 6 h to four stimuli, including LPS, Pam_3_CSK_4_, R848, and a human seasonal influenza A virus. The DICE and Immvar datasets were composed of all Europeans, aged between 18 and 61 years (median age of 27 years) and with a 1-to-1 sex ratio between males and females. The EvoImmunoPop dataset was composed of only males, aged 20–50, and balanced between African and European ancestry, with all individuals recruited in Belgium.

### Mapping of RNA-seq data across different cell types

Original RNA-seq reads were aligned with the human genome (hg19/GRCh37), using STAR^35^, version 2.5.2b 51, with the following alignment parameters: outSAMtype, BAM; SortedByCoordinate; outSAMstrandField, intronMotif; outFilterMultimapNmax, 10; outFilterMultimapScoreRange, 1; alignSJDBoverhangMin, 1; sjdbScore, 2; alignIntronMin, 20; and alignSJoverhangMin, 8. The resulting sorted BAM files were converted into bedgraph formats using bedtools, version 2.17.0.

### Genotype imputation

We imputed variants into the 1000 Genomes reference panel using SHAPEIT^36^ and IMPUTE2^37^. We filtered out variants with an ‘info score’ <0.9, a multiallelic, Hardy–Weinberg equilibrium threshold *P* < 1 × 10^−5^, and a MAF < 1% in Europeans/Africans of the 1000 Genomes reference panel and indels. We include the top 3 principal components (PCs) as known genotypic covariates, while for the traits with genomic control lambda are larger than 1.05, the top 10 PCs were included as covariates.

### DaPars2 analyses

We applied DaPars2^23^ to multiple RNA-seq data. For the quantification step, the proximal polyA sites were identified by joint analyzing all samples. We further calculated the percentage of distal polyA site usage index (PDUI) for each transcript in each sample. We required that the average normalized reads for each RefSeq 3′UTR region be > 20; otherwise, the PDUI for this transcript was assigned as a missing value, “NA”. For a given tissue, transcripts with missing values in > 50% of individuals were removed.

### Identifying differentially regulated APA events

Differentially regulated APA genes were assessed using the two-sided Wilcoxon rank test, and P values were corrected for multiple testing across all conditions using the Benjamini-Hochberg false discovery rate (FDR) method. We defined the significant APA events as when the PDUI differences > 0.1 and FDR < 0.05.

### Immune 3′aQTL mapping for each cell type

SNPs with a minor allele frequency of < 0.01 were filtered, and at least 10 counts per allele were required. For each tissue, we then used Matrix eQTL^38^ with a standard additive linear model to test associations for SNPs within a linear regression framework.

Permutation analysis was conducted to identify significant 3′aQTL-associated gene pairs. Individual labels were randomly sampled 1000 times, and the minimum *P* value for each SNP and gene was recorded after each 3′aQTL mapping. These empirical *P* values were adjusted using the *q* value R package. Genes with a *q*-value of <0.05 were considered to be significant APA genes. All APA gene-associated 3′aQTLs were subsequently identified with the FDR set to 5%. We tested each ancestry groups individually and the effect of merging both ancestry groups and found that ancestry group had a minimal impact on APA variations; thus, we merged the populations in this analysis, and ancestry groups (AFB, EUB) were included as known covariates for 3′aQTL mapping.

### Estimating immune 3′aQTL heritability

The genome-wide complex trait analysis-genome-based restricted maximum likelihood (GCTA-GREML)^27^ program was used to estimate the heritability of immune 3′aQTLs.

### Immune 3′aQTL sharing and specificity analyses among tissues

3′aQTL sharing and specificity among tissues were analyzed using multivariate adaptive shrinkage (mash)^39^. Briefly, we converted 3′aQTL association statistics to mash formats. Lead 3′aQTLs and random SNP sets for each APA gene were extracted from each tissue to calculate mash priors. Prior covariance matrices were inferred via Empirical Bayes matrix factorization and implemented in factors and loadings by adaptive shrinkage (FLASH); the multivariate 3′aQTL model was constructed using mash. Posterior effect sizes were computed by applying the trained model to the lead 3′aQTLs sets. The mash method aims to elucidate the heterogeneity of 3′aQTL effect sizes across tissues. It also provides a local false sign rate to calculate the probability that the estimated effect size has the incorrect sign.

### Determining overlap between immune 3′aQTLs and eQTLs

3′aQTLs and eQTLs were considered to overlap when the lead 3′aQTL (top APA-associated SNPs) or its LD tag (R^2^ $\ge$ 0.8) mapped with the lead eQTL (top expression-associated SNPs) for the same gene.

### Collection of GWAS summary statistics

We collected 52 GWAS summary statistics from previous published literatures. These traits include 40 autoimmune and blood-related traits and 12 other traits, which is listed in Supplementary Data 7.

### Co-localization analyses

We applied a Bayesian co-localization approach to identify GWAS signals that could exhibit the same genetic effect with immune 3′aQTLs and eQTLs using coloc R (version 5.1.0) package^32^. The eQTL summary statistics are obtained from eQTL catalogue^40^. We used the default coloc priors for Bayesian co-localization analysis, in which the prior was assigned 1 × 10^−4^ for representing the probability that the SNP was associated with either trait or 3′aQTL/eQTL, and $1\times {10}^{-5}$ ${{{{{\rm{for\; representing}}}}}}$ the probability that the SNP was associated with both trait and 3′aQTL/eQTL. For each GWAS trait, we extracted the GWAS SNPs with a *P*-value < 5 × 10^−8^ and located at least 1 Mb away from more significant variants. The co-localized signals were searched within a surrounding region of 100 kb of GWAS SNPs. Five posterior probabilities (PPs) were calculated for the colocalization analysis using all variants in the region of interest. PP0 represents the null model of no association. PP1 and PP2 represent the probability that causal genetic variants are either associated with disease signals only or 3′aQTL only. PP3 represents the probability that the genetic effects of disease signals and 3′aQTL/eQTL are independent, and PP4 represents the probability that disease signals and 3′aQTL/eQTL share causal SNPs. The genes were defined as co-localization events if PP4$\ge$0.75 and PP4/(PP4 + PP3)$\ge \,$0.9 and with significant 3′aQTLs. Region visualization plots were constructed using LocusZoom^41^. LD associations between reference SNPs and 3′aQTLs/eQTL were calculated using PLINK^42^ v2.0.

### Summary data-based Mendelian randomization (SMR) analysis

We performed SMR analysis using SMR^43^ version 1.0.2 on the GWAS summarized data and immune 3′aQTLs to test for a joint pleiotropic association between APA and each trait due to a shared causal variant. The lead 3′aQTL was used to estimate the APA effect on the outcome. We used the following settings: --maf 0.01, --thread-num 10, --diff-freq 1. We considered SMR *p*-value < 3.5e−9 to be significant by taking into account of the number of genetic regions, the number of traits and the number of cell types. To distinguish pleiotropy/causation from linkage, we also used the heterogeneity statistic and required that p_heidi > 0.05 to exclude genes due to linkage.

### Reporting summary

Further information on research design is available in the Nature Portfolio Reporting Summary linked to this article.

### Supplementary information

Supplementary Information

Description of Additional Supplementary Files

Supplementary Data 1-9

Reporting Summary

## Data Availability

The human reference genome hg19 and gene annotations were obtained from the UCSC genome browser (https://genome.ucsc.edu). Genome-wide SNP genotype, whole-exome sequencing, and RNA-sequencing data of EvoImmunoPop project used are deposited in the European Genome-phenome Archive (EGA) under accession code EGAS00001001895. Genotype and RNA-sequencing data from the BLUEPRINT project are deposited in EGA under accession code EGAD00001002671, EGAD00001002674 and EGAD00001002675. In accordance with the General Data Protection Regulation (GDPR) in force in the European Union, the aforementioned data from EGA can be accessed only from the institutional data repository after authorization by the relevant Data Access Committee (DAC). The DAC ensures that data access and use is authorized for academic research relating to the variability of the human immune response, as defined in the informed consent signed by research participants. Further details of the application procedure can be obtained from EGA. Genotype and RNA-sequencing data from the DICE project are deposited in the Genotype and Phenotypes (dbGaP) database under the accession code phs001703.v4.p1. Genotype and RNA-sequencing data from the ImmVar project are deposited in dbGaP under accession code phs000815.v2.p1. All relevant data from dbGaP are available under controlled access. The data described in this study are freely available for querying, visualizing, and downloading at http://bioinfo.szbl.ac.cn/immune_aqtl/index.php, a website portal dedicated to immune 3′aQTLs. Immune 3′aQTLs are also freely available in Synapse (accession number: syn51899720, 10.7303/syn51899720), ensuring the availability of a wide range of information related to this study. List of APA genes and 3′aQTLs identified in this study is provided in Supplementary Data 1. Immune 3’aQTLs specific to the major immune groups are provided in Supplementary Data 2. List of significant APA events comparing the stimulated conditions with baseline conditions is provided in Supplementary Data 3. The data for response 3’aQTLs generated in this study are provided in Supplementary Data 4–6. List of human diseases and complex traits examined in this study is provided in Supplementary Data 7. The co-localization data generated in this study are provided in Supplementary Data 8. The SMR data generated in this study are provided in Supplementary Data 9.
