# Supplementary material for: Immune-response 3′UTR alternative polyadenylation quantitative trait loci contribute to variation in human complex traits and diseases
Source: Nat Commun. 2023 Dec 15;14:8347. doi: 10.1038/s41467-023-44191-1 (PMC10724249; doi:10.1038/s41467-023-44191-1)
Supplement: Supplementary file 1 — Supplementary Information [file 41467_2023_44191_MOESM1_ESM.pdf]

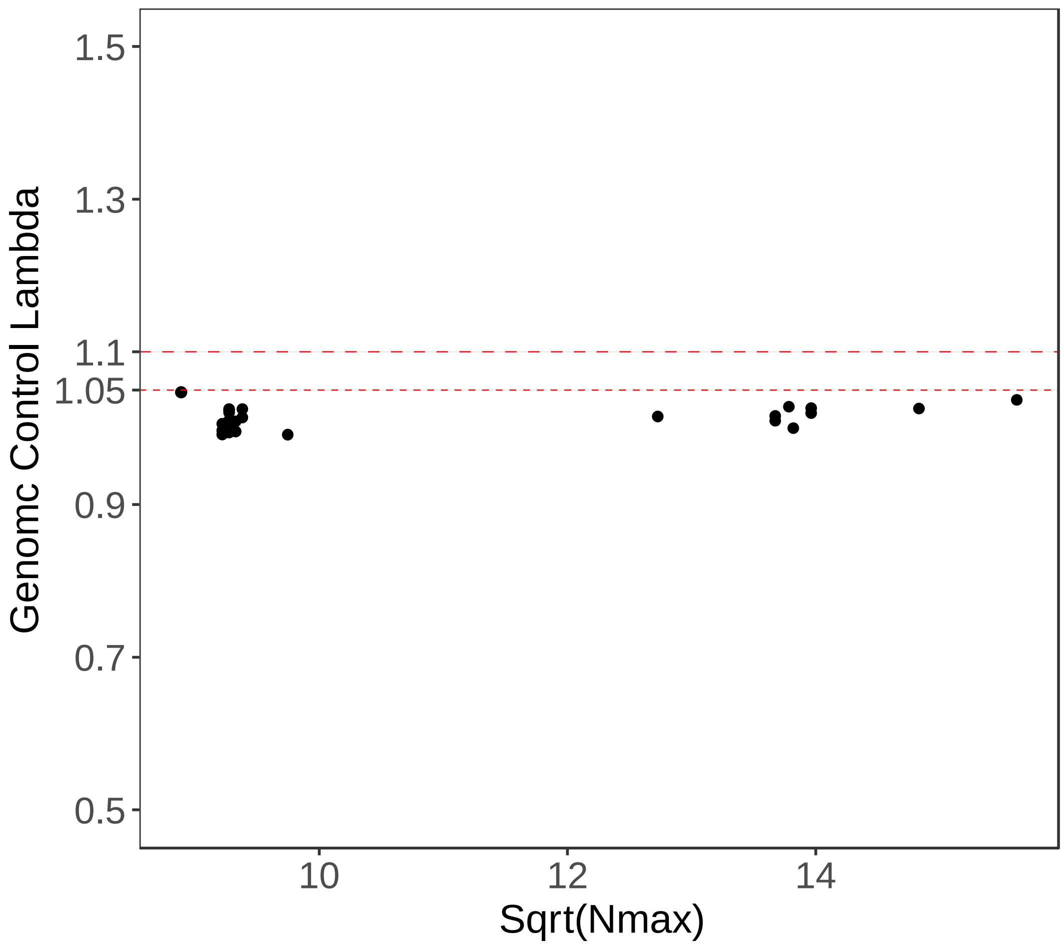


Supplementary Fig. 1. The Lambda-N plot shows the association of sample size with genomic control lambda value. The red line indicates the genomic control lambda value is 1.05 and 1.1.

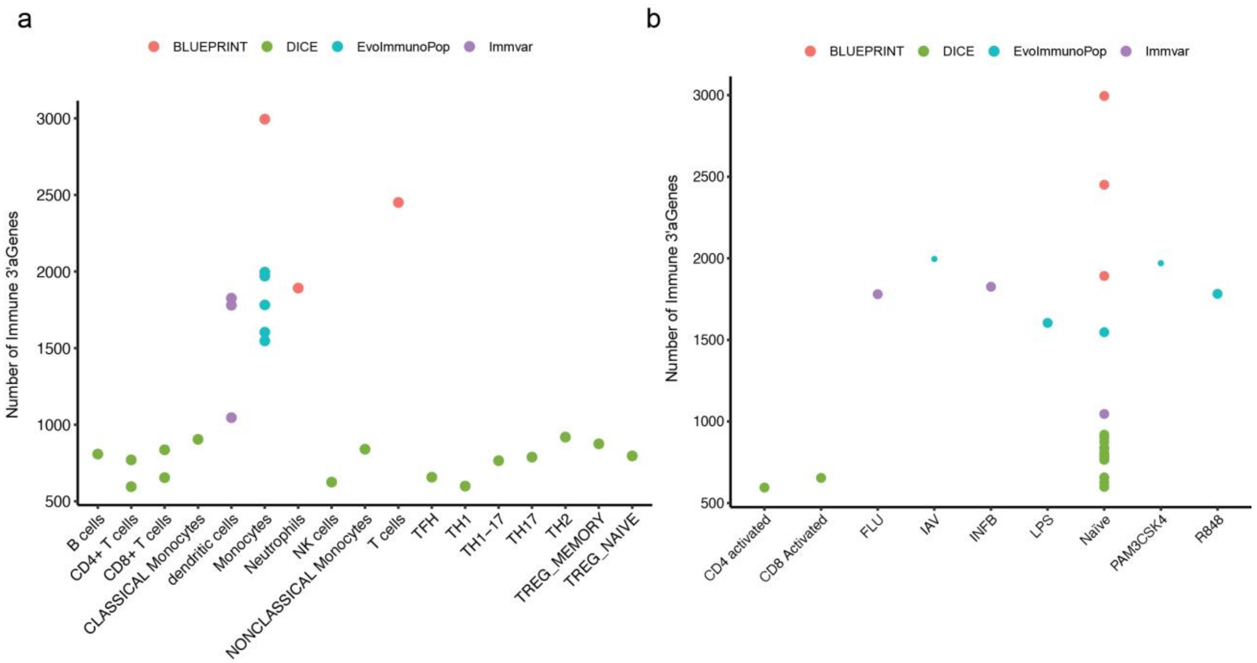


Supplementary Fig. 2. Scatter plot depicting the distribution of immune 3'aGenes across various immune cell types. (a) The distribution of the number of immune 3'aGenes with immune cell types. (b) The distribution of the number of immune 3'aGenes with stimulation condition. The dots with different colors represent different datasets.

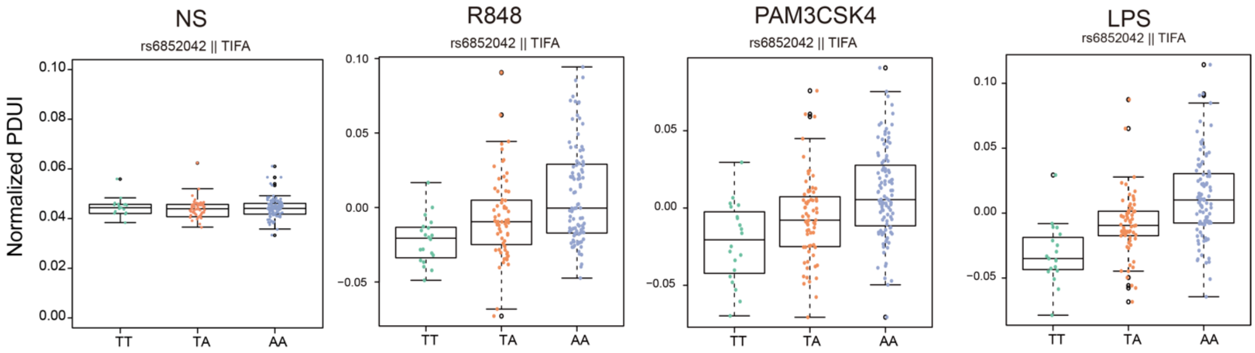


Supplementary Fig. 3. Example of response 3’aQTL where the 3’aQTL rs6852042 impacts a canonical polyA site in NS, R848, $PAM_3CSK_4$, and LPS conditions. The center horizontal lines of the box plot show the median values and the boxes span from the 25th to the 75th percentile (n=200).

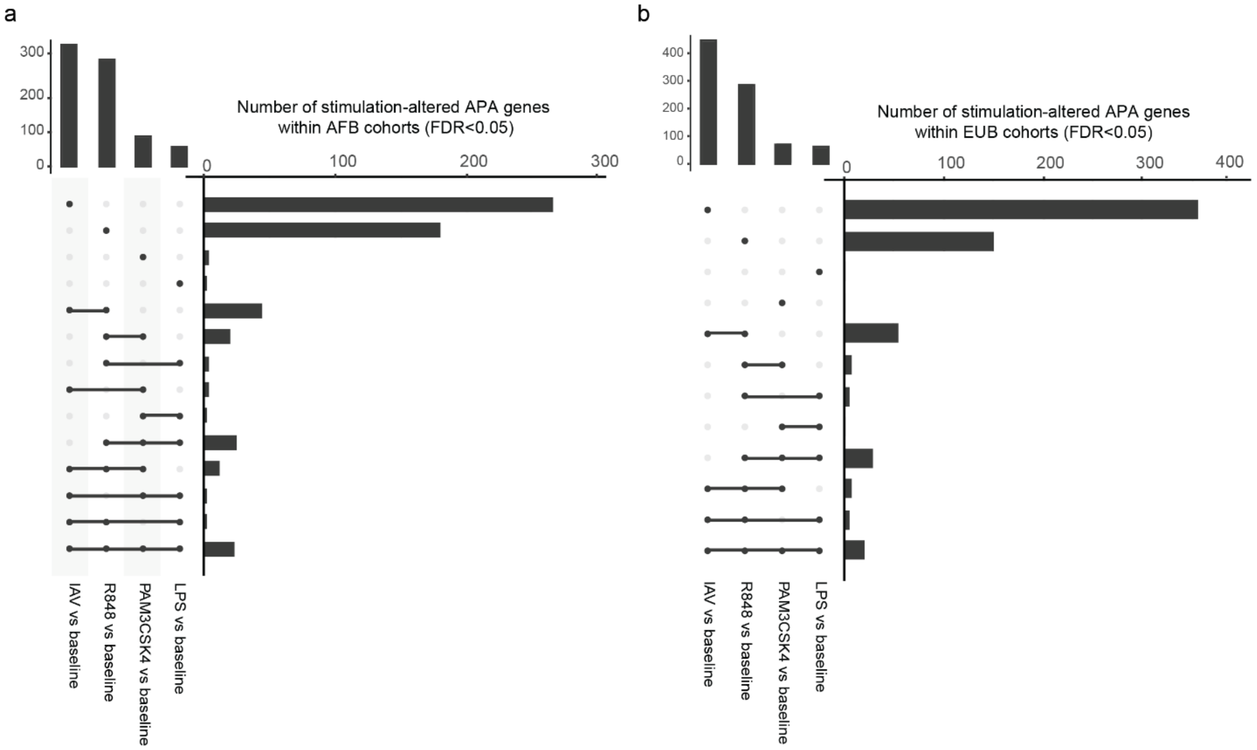


Supplementary Fig. 4. Number of immune-stimulated APA genes compared to control in AFB cohorts and EUB cohorts. (a) Number of immune-stimulated APA genes within AFB cohorts (FDR<0.05). (b) Number of immune-stimulated APA genes within EUB cohorts (FDR<0.05).

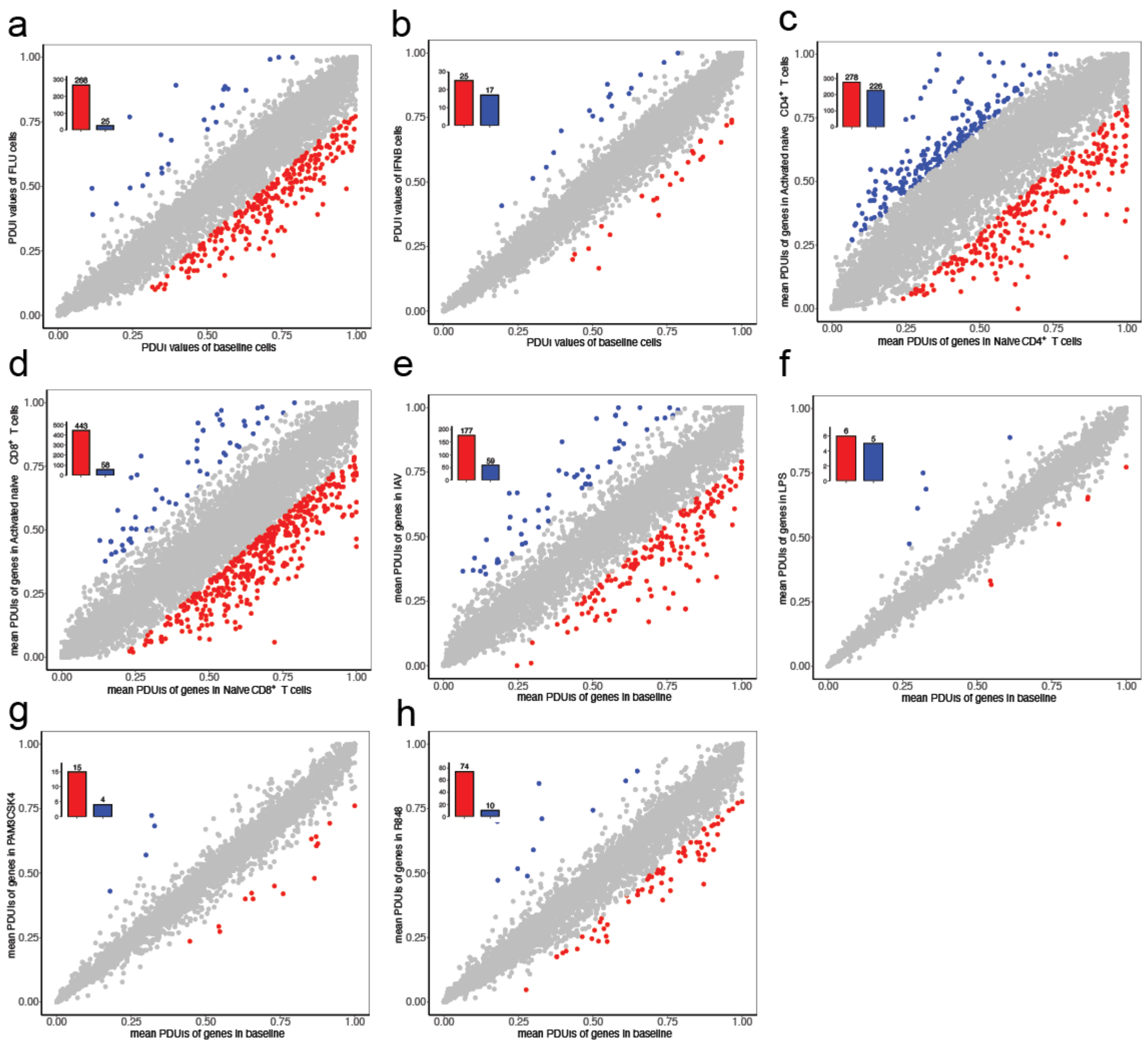


Supplementary Fig. 5. Scatterplot of PDUI values in baseline and stimulation conditions. (a) Scatterplot of PDUI values in baseline and FLU cells. (b) Scatterplot of PDUI values in baseline and IFNB cells. (c) Scatterplot of PDUI values in naive $CD4^+$ T cells and activated $CD4^+$ T cells. (d) Scatterplot of PDUI values in naive $CD8^+$ T cells and activated $CD8^+$ T cells. (e) Scatterplot of PDUI values in baseline and IAV cells. (f) Scatterplot of PDUI values in baseline and LPS cells. (g) Scatterplot of PDUI values in baseline and $Pam_3CSK_4$ cells. (h) Scatterplot of PDUI values in baseline and R848 cells. The number of transcripts with PDUIs differences ≥ 0.2 between baseline and stimulation through the threshold of p-value ≤0.05 are listed in the top left corn by bar plot. Red color represents 3'UTR shortening in stimulation conditions, and blue color represents 3'UTR lengthening in stimulation conditions.

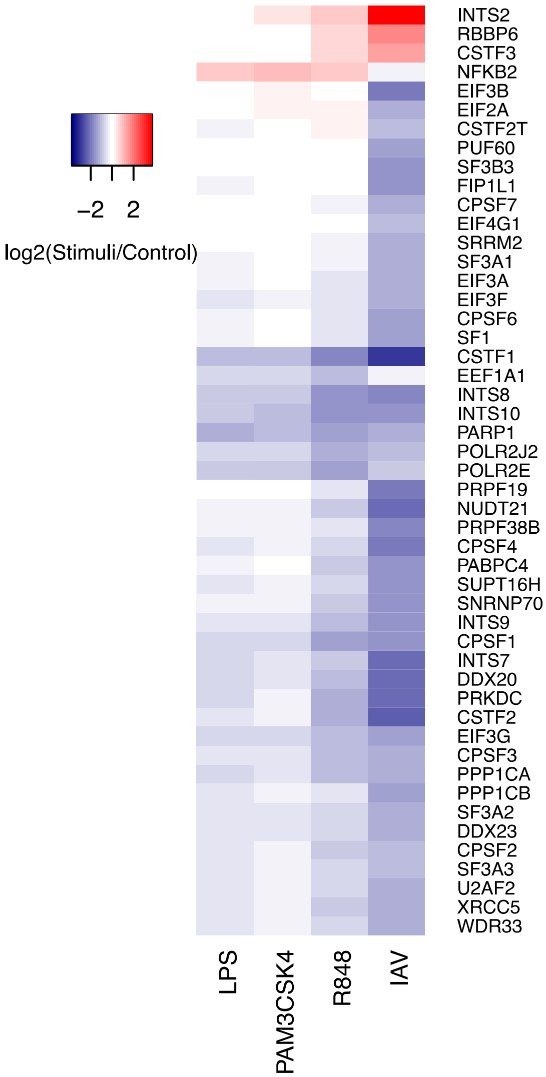


Supplementary Fig. 6. Heatmap of expression of APA regulators. The X-axis is stimulation conditions (LPS, $PAM_3CSK_4$, R848, IAV) for the EvoImmunoPop dataset, and the Y-axis is APA regulators. The color represents log2(the fold change of the expression values in stimulations compared with the baseline).

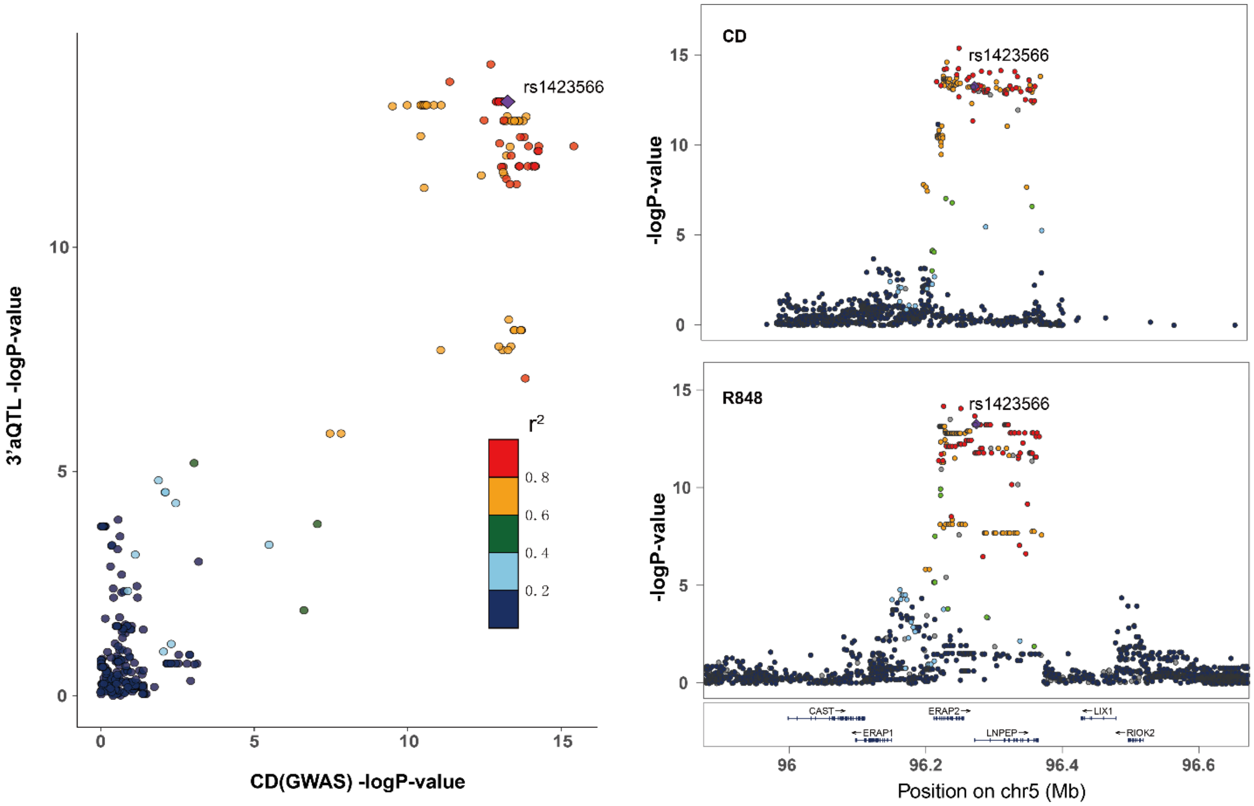


Supplementary Fig. 7. Colocalization plot of response 3'aQTLs for *ERAP2* that co-localized with Crohn's disease for the R848 stimulation conditions.
