# Supplementary material for: Immune-response 3′UTR alternative polyadenylation quantitative trait loci contribute to variation in human complex traits and diseases
Source: Nat Commun. 2023 Dec 15;14:8347. doi: 10.1038/s41467-023-44191-1 (PMC10724249; doi:10.1038/s41467-023-44191-1)
Supplement: Supplementary file 2 — Description of Additional Supplementary Files [file 41467_2023_44191_MOESM2_ESM.pdf]

Description of Additional Supplementary Files

File name: Supplementary Data 1
Description: List of APA genes and 3′aQTLs identified in this study.
APA genes and 3′aQTLs identified in this study. Columns include: 1) cell type name, 2) study, 3) cell types, 4) number of samples per tissue, 5) number of 3′aQTLs with FDR ≤ 0.05, and 6) number of 3′aQTL-associated genes (3′aQTLs) with q-value ≤ 0.05.

File name: Supplementary Data 2
Description: Immune 3′aQTLs are specific for major immune groups.
List of the local false sign rate (LFSR) value for major immune groups. The 3′aQTLs with LFSR < 0.05 indicate that the 3′aQTLs' effect is significant in the cell type.

File name: Supplementary Data 3
Description: List of significant APA events comparing the stimulated conditions with baseline conditions.
List of each significant APA event when comparing the stimulated conditions (IAV, LPS, Pam3CSK4, R848) with the baseline conditions.

File name: Supplementary Data 4
Description: Response 3′aQTLs identified in the EvoImmunoPop datasets.
List of the LFSR value for each response's 3′aQTLs. The 3′aQTLs with LFSR < 0.05 indicate that the 3′aQTLs' effect is significant in the cell type.

File name: Supplementary Data 5
Description: Response 3′aQTLs identified in dendritic cells.
List of the LFSR value for each response's 3′aQTLs. The 3′aQTLs with LFSR < 0.05 indicate that the 3′aQTLs' effect is significant in the cell type.

File name: Supplementary Data 6
Description: Response 3′aQTLs identified in ImmVar T cells.
List of the LFSR value for each response's 3′aQTLs. The 3′aQTLs with LFSR < 0.05 indicate that the 3′aQTLs' effect is significant in the cell type.

File name: Supplementary Data 7
Description: List of human diseases and complex traits examined in this study.
List of the different complex human traits and diseases examined, with downloaded URLs provided.

File name: Supplementary Data 8
Description: List of co-localizing human trait GWAS signals and immune 3′aQTLs.
List of the genes for which the associated 3′aQTLs/eQTLs co-localized with GWAS signals. PP0 indicates the null model of no association. PP1 and PP2 indicate the probability that causal variants are either associated with disease signals only or with 3′aQTL only, respectively. PP3 indicates the probability that the genetic effects of disease signals and 3′aQTLs are independent. PP4 indicates the probability that disease signals and 3′aQTLs share causal SNPs.

File name: Supplementary Data 9
Description: List of significant SMR results.
List of each significant APA event identified from SMR analysis results.
