# Supplementary material for: Immune-response 3′UTR alternative polyadenylation quantitative trait loci contribute to variation in human complex traits and diseases
Source: Nat Commun. 2023 Dec 15;14:8347. doi: 10.1038/s41467-023-44191-1 (PMC10724249; doi:10.1038/s41467-023-44191-1)
Supplement: Supplementary file 4 — Reporting Summary [file 41467_2023_44191_MOESM4_ESM.pdf]

nature portfolio

Corresponding author(s): Lei Li and Wei Li

Last updated by author(s): Nov 15, 2023

Reporting Summary

Nature Portfolio wishes to improve the reproducibility of the work that we publish. This form provides structure for consistency and transparency in reporting. For further information on Nature Portfolio policies, see our Editorial Policies and the Editorial Policy Checklist.

Statistics

For all statistical analyses, confirm that the following items are present in the figure legend, table legend, main text, or Methods section.

n/a	Confirmed	
☐	☒	The exact sample size (*n*) for each experimental group/condition, given as a discrete number and unit of measurement
☐	☒	A statement on whether measurements were taken from distinct samples or whether the same sample was measured repeatedly
☐	☒	The statistical test(s) used AND whether they are one- or two-sided<br>*Only common tests should be described solely by name; describe more complex techniques in the Methods section.*
☐	☒	A description of all covariates tested
☐	☒	A description of any assumptions or corrections, such as tests of normality and adjustment for multiple comparisons
☐	☒	A full description of the statistical parameters including central tendency (e.g. means) or other basic estimates (e.g. regression coefficient) AND variation (e.g. standard deviation) or associated estimates of uncertainty (e.g. confidence intervals)
☐	☒	For null hypothesis testing, the test statistic (e.g. *F*, *t*, *r*) with confidence intervals, effect sizes, degrees of freedom and *P* value noted<br>*Give P values as exact values whenever suitable.*
☒	☐	For Bayesian analysis, information on the choice of priors and Markov chain Monte Carlo settings
☒	☐	For hierarchical and complex designs, identification of the appropriate level for tests and full reporting of outcomes
☐	☒	Estimates of effect sizes (e.g. Cohen's *d*, Pearson's *r*), indicating how they were calculated

Our web collection on statistics for biologists contains articles on many of the points above.

Software and code

Policy information about availability of computer code

Data collection	No software was used for data collection.
Data analysis	STAR v2.5.2b, PEER v1.3, GCTA v1.93, bcftools v1.3, Matrix eQTL v2.1.0, SuSiE (https://github.com/stephenslab/susieR), R 3.4.0, Ldsr v1.0.1, Coloc v3.2-1, bedtools v2.17.0, Coloc v3.2-1 with coloc R v5.1.0, SMR v1.0.2, PLINK v2.0, The open-source DaPars v2 program is freely available at https://github.com/3UTR/DaPars2.

For manuscripts utilizing custom algorithms or software that are central to the research but not yet described in published literature, software must be made available to editors and reviewers. We strongly encourage code deposition in a community repository (e.g. GitHub). See the Nature Portfolio guidelines for submitting code & software for further information.

Data

Policy information about availability of data

All manuscripts must include a data availability statement. This statement should provide the following information, where applicable:

- Accession codes, unique identifiers, or web links for publicly available datasets
- A description of any restrictions on data availability
- For clinical datasets or third party data, please ensure that the statement adheres to our policy

The human reference genome hg19 and gene annotations were obtained from the UCSC genome browser (https://genome.ucsc.edu). Genome-wide SNP genotype, whole-exome sequencing, and RNA-sequencing data of EvoImmunoPop project used are deposited in the European Genome-phenome Archive (EGA) under accession code EGAS00001001895 (https://ega-archive.org/studies/EGAS00001001895). Genotype and RNA-sequencing data from the BLUEPRINT project are deposited in EGA under accession code EGAD00001002671 (https://ega-archive.org/datasets/EGAD00001002671), EGAD00001002674 (https://ega-archive.org/datasets/EGAD00001002674) and EGAD00001002675 (https://ega-archive.org/datasets/EGAD00001002675). Genotype and RNA-sequencing data from the DICE

project are deposited in the Genotype and Phenotypes (dbGaP) database under the accession code phs001703.v4.p1 (https://www.ncbi.nlm.nih.gov/projects/gap/cgi-bin/study.cgi?study_id=phs001703.v4.p1). Genotype and RNA-sequencing data from the ImmVar project are deposited in dbGaP under accession code phs000815.v2.p1 (https://www.ncbi.nlm.nih.gov/projects/gap/cgi-bin/study.cgi?study_id=phs000815.v2.p1). The data described in this study are freely available for querying, visualizing, and downloading at http://bioinfo.szbl.ac.cn/immune_aqtl/index.php, a website portal dedicated to immune 3'aQTLs. Immune 3'aQTLs are also freely available in Synapse (accession number: syn51899720, doi: doi.org/10.7303/syn51899720), ensuring the availability of a wide range of information related to this study.

Field-specific reporting

Please select the one below that is the best fit for your research. If you are not sure, read the appropriate sections before making your selection.

☒ Life sciences ☐ Behavioural & social sciences ☐ Ecological, evolutionary & environmental sciences

For a reference copy of the document with all sections, see nature.com/documents/nr-reporting-summary-flat.pdf

Life sciences study design

All studies must disclose on these points even when the disclosure is negative.

Sample size	Sample size was determined based on the availability of existing DICE, Blueprint, Immvar and EvoImmunoPop data.
Data exclusions	We removed TCEL cell type from ImmVar dataset.
Replication	In EvoImmunoPop dataset, the reproducibility of RNA-Seq profiles was evaluated by technical and biological replicates on seven independent individuals (4 Africans and 3 Europeans) across the five experimental conditions. Other experiments has been performed independently with biological triplicates.
Randomization	The samples have been assigned randomly at the beginning of experiments. In the EvoImmunoPop dataset, a balanced design was implemented, wherein each experimental batch consisted of three randomly selected individuals of European descent and three randomly selected individuals of African descent. Stimulated and resting monocyte samples were pooled across all batches and randomized before library preparation. Sequencing libraries were prepared in groups of approximately 12 randomly selected samples, and within each lane, samples were pooled in groups of 6, including 3 randomly selected individuals of European descent and 3 of African descent.
Blinding	The bioinformatics analyses have been corroborated with blinded wet lab experiments.

Reporting for specific materials, systems and methods

We require information from authors about some types of materials, experimental systems and methods used in many studies. Here, indicate whether each material, system or method listed is relevant to your study. If you are not sure if a list item applies to your research, read the appropriate section before selecting a response.

Materials & experimental systems

n/a	Involved in the study	
☒	☐	Antibodies
☒	☐	Eukaryotic cell lines
☒	☐	Palaeontology and archaeology
☒	☐	Animals and other organisms
☒	☐	Human research participants
☒	☐	Clinical data
☒	☐	Dual use research of concern

Methods

n/a	Involved in the study	
☒	☐	ChIP-seq
☒	☐	Flow cytometry
☒	☐	MRI-based neuroimaging
